# Pharmacist-led intervention for older people with atrial fibrillation in long-term care (PIVOTALL study): a randomised pilot and feasibility study

**DOI:** 10.1186/s12877-023-04527-4

**Published:** 2024-01-16

**Authors:** Leona A. Ritchie, Peter E. Penson, Asangaedem Akpan, Gregory Y. H. Lip, Deirdre A. Lane

**Affiliations:** 1 Liverpool Centre for Cardiovascular Science, William Henry Duncan Building, University of Liverpool, Liverpool, L7 8TX UK; 2 Department of Cardiovascular and Metabolic Medicine, Institute of Life Course and Medical Sciences, University of Liverpool, Liverpool, L7 8TX UK; 3 Clinical Pharmacy and Therapeutics Research Group, School of Pharmacy and Biomolecular Sciences, Liverpool John Moores University, Liverpool, L3 3AF UK; 4 Musculoskeletal and Ageing Science, Institute of Life Course and Medical Sciences, University of Liverpool, Liverpool, L7 8TX UK; 5 Liverpool University Hospitals NHS Foundation Trust, Liverpool, L9 7AL UK; 6 Liverpool Centre for Cardiovascular Science, University of Liverpool, Liverpool John Moores University and Liverpool Heart and Chest Hospital, Liverpool, UK; 7 Danish Center for Health Services Research, Department of Clinical Medicine, Aalborg University, Aalborg, Denmark

**Keywords:** Atrial fibrillation, Care homes, Older people, Pharmacists, Feasibility study, Integrated care, Medication optimisation

## Abstract

**Background:**

Older care home residents are a vulnerable group of people with atrial fibrillation (AF) at high risk of adverse health events. The Atrial Fibrillation Better Care (ABC: Avoid stroke; Better symptom management; Cardiovascular and other comorbidity management) pathway is the gold-standard approach toward integrated AF care, and pharmacists are a potential resource with regards to its’ implementation. The aim of this study was to determine the feasibility of pharmacist-led medicines optimisation in care home residents, based on the ABC pathway compared to usual care.

**Methods:**

Individually randomised, prospective pilot and feasibility study of older (aged ≥ 65 years) care home residents with AF (ISRCTN14747952); residents randomised to ABC pathway optimised care versus usual care. The primary outcome was a description of study feasibility (resident and care home recruitment and retention). Secondary outcomes included the number and type of pharmacist medication recommendations and general practitioner (GP) implementation.

**Results:**

Twenty-one residents were recruited and 11 (mean age [standard deviation] 85.0 [6.5] years, 63.6% female) were randomised to receive pharmacist-led medicines optimisation. Only 3/11 residents were adherent to all three components of the ABC pathway. Adherence was higher to ‘A’ (9/11 residents) and ‘B’ (9/11 residents) components compared to ‘C’ (3/11 residents). Four ABC-specific medicines recommendations were made for three residents, and two were implemented by residents’ GPs. Overall ABC adherence rates did not change after pharmacist medication review, but adherence to ‘A’ increased (from 9/11 to 10/11 residents). Other ABC recommendations were inappropriate given residents’ co-morbidities and risk of medication-related adverse effects.

**Conclusions:**

The ABC pathway as a framework was feasible to implement for pharmacist medication review, but most residents’ medications were already optimised. Low rates of adherence to guideline-recommended therapy were a result of active decisions not to treat after assessment of the net risk–benefit.

**Supplementary Information:**

The online version contains supplementary material available at 10.1186/s12877-023-04527-4.

## Introduction

There are no estimates of the number of people living in long-term care institutions across Europe [1], but it is forecast that many European countries need to create more long-term care beds to cope with increasing demand [2]. In the United Kingdom (UK), it is estimated that over 360,000 people aged 65 years and older live in long-term care [3]. This includes people living in residential and nursing homes, collectively referred to as care homes throughout this paper. Optimal management of atrial fibrillation (AF) in older care home residents is critical to reduce the risk of adverse health outcomes that have a detrimental impact on resident quality of life. The prevalence and incidence of AF increases with advancing age [4, 5], and a recent estimate of prevalence was 17.4% in older care home residents in Wales, UK, between 2010–2018 [6]. Another smaller scale study in France reported AF prevalence as 10.1% in 10,660 residents across 104 nursing homes [7]. There is a paucity of European and global care home data.

Care home residents with AF have a significantly higher risk of stroke and cardiovascular hospitalisation compared to residents without AF, even in the context of advanced frailty and limited life expectancy [6]. The risk of all-cause and cardiovascular mortality is also significantly higher in care home residents with AF compared to those without AF [6]. The Atrial Fibrillation Better Care (ABC: A, Avoid stroke; B, Better symptom management; C, Cardiovascular and other co-morbidity optimisation) pathway [8] is recommended as the gold-standard management approach to deliver integrated AF care [9, 10]. Available evidence lends support to implementation of the ABC pathway in care home residents. Three observational studies reported a significant association between adherence to all three ABC pathway components and a lower risk of adverse health outcomes in people with frailty [11], multiple chronic conditions [12, 13], polypharmacy [12, 13] and prior hospitalisation [12]. However, to date, no study has prospectively tested the feasibility of implementing the ABC pathway in older care home residents, nor collected data on person-centred outcomes.

In the UK, there is an increasing focus on pharmacy services across community, hospital and general practice to support care homes after recent investments by National Health Service (NHS) England [14] and the contractual requirement for networks of general practices to identify and prioritise people who would benefit from a structured medication review [15]. Pharmacists have been identified as a potential resource in regard to AF management, with evidence to suggest they can help to operationalise and implement ABC adherent care within primary and secondary care [16].

## Methods

### Study design

The pharmacist-led intervention for AF in long-term care (PIVOTALL) study was a single-blinded, individually randomised pilot and feasibility study, consistent with a consensus-agreed conceptual framework proposed for defining pilot and feasibility studies [17]. It was conducted in accordance with the Consolidated Standards of Reporting Trials (CONSORT) statement extension for pilot and feasibility studies [18]. Ethical approval was obtained from Health and Care Research Wales (08/06/2020) and the Health Research Authority (15/06/2020) after favourable review by Wales Research Ethics Committee 4 (ref: 20/WA/0164). The study was prospectively registered on 02/10/2020 with the International Standard Randomised Controlled Trial Number Registry (ISRCTN14747952) [19]. A formal sample size calculation was not performed to determine the target sample size, but the study aimed to recruit a minimum of 50 residents in keeping with previously reported sample sizes per group for pilot studies (median of 30; range 8 to 114 participants) and feasibility studies (median of 36; range 10 to 300 participants) registered on the UK Clinical Research Network [20].

#### Study setting

Care homes (including general residential, general nursing, nursing Elderly Mentally Infirm (EMI) and residential EMI homes) in Liverpool, UK and Sefton, UK. Residential homes provide a supported living environment with personal care assistance. Nursing homes provide a higher level of medical care with the support of qualified nurses. EMI homes refer to nursing or residential homes for people with dementia.

#### Study participants

Participants were older residents (aged ≥ 65 years) of care homes with a diagnosis of AF. People who were (1) receiving end-of-life care, (2) non-English speaking, (3) diagnosed with aphasia or (4) identified as short-stay care home residents (expected stay < 6 months), were excluded. Participants who did not have capacity were only eligible for inclusion if they had a Lasting Power of Attorney (LPA) for Health and Welfare who could consent on their behalf.

#### Identification and recruitment of care homes and residents

All care homes within Liverpool and Sefton Council and registered with the Care Quality Commission were eligible to take part. Most care homes were approached about the study during virtual meetings, and introductions were predominantly facilitated by a consultant geriatrician (AA) who was part of the PIVOTALL research team, or by medicines management pharmacists working within the care home setting. A senior representative from each care home was required to sign a Site agreement and a Participant Identification Centre (PIC) agreement in order to register as a participating site (Supplement 1, Figure S1.1). General practices providing medical support to participating care homes were asked to sign a PIC agreement to authorise them to act as PIC sites. In the event that a care home was supported by multiple general practices, consent was sought from the practice where most of the residents were registered. The responsible general practitioner (GP) gave authority for a nominated facilitator (external to the research team) to run a search on an electronic health record system (EMIS; Egton Medical Information Systems) used by primary care in the UK to identify eligible care home residents (Supplement 1, Figure S1.1). To maximise recruitment, where possible, the EMIS search was repeated after six months. The nominated facilitator provided a list of resident names identified from the EMIS search, along with invitation-to-study letters, to the care home. Care home staff approached residents with capacity (or the LPA for Health and Welfare of residents without mental capacity) and provided invitation-to-study letters before obtaining their consent to be contacted by a researcher (Supplement 1, Figure S1.1). After one week, a researcher contacted the care home and asked to be provided with the name(s) of the resident(s), or their LPA for Health and Welfare (if applicable) who had not declined to being contacted by the research team. Potential participants (or their LPA for Health and Welfare) were contacted by telephone or visited and provided with verbal and written information about the study. Written consent was obtained from residents with capacity who agreed to take part in the study. Health and Welfare LPAs of residents without capacity were asked to sign a consultee declaration if they agreed to their friend/relative taking part (Supplement 1, Figure S1.1). After recruitment, residents were individually randomised to the intervention or usual care group using an electronic randomisation system on REDCap, stratified by site (care home). The randomisation system was developed by a trial statistician from Liverpool Clinical Trials Centre who was independent to the PIVOTALL researchers. An EMIS login was issued by general practices acting as PIC sites to permit researcher access to residents’ electronic health records.

#### Treatment groups

All residents’ GPs received a letter to inform them if one of their patients was recruited and which treatment group (intervention or usual care) they were allocated to. Residents in the intervention group received a pharmacist-led medication review by a researcher (pharmacist LAR) based on the ABC pathway framework. Pharmacist (LAR) was not established as part of the residents’ usual healthcare team but had been introduced (remotely) to the residents’ GPs who would receive medicines recommendations made as part of the study.

Adherence to the ABC pathway was assessed in residents allocated to the intervention group before and after the medication review. Adherence was defined as residents prescribed anticoagulation (where indicated) for stroke prevention: ‘A’, with optimal management of their AF symptoms: ‘B’, and other cardiovascular co-morbidities: ‘C’. Comprehensive definitions for each pathway component are shown in Table 1. Where appropriate, medication recommendations were made to residents’ GPs using a standardised medication review form uploaded to the resident’s electronic health records using the EMIS platform. Residents in the intervention group who were not A, B and C adherent at baseline had recommendations made after the review. Medication recommendations were not made at follow-up, irrespective of ABC adherence.

**Table 1:**
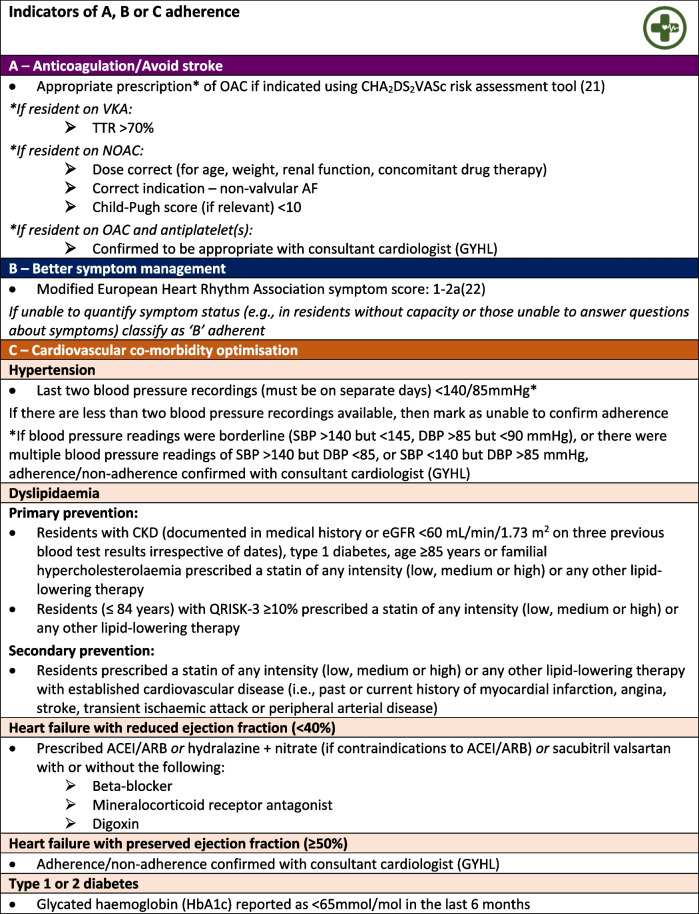
Definitions of Atrial Fibrillation Better Care (ABC) pathway adherence used as the framework for the pharmacist-led medication review [21, 22]

*ACEI* angiotensin converting enzyme inhibitor, *ARB* angiotensin receptor blocker, *CKD* chronic kidney disease, *DBP* diastolic blood pressure, *eGFR* estimated glomerular filtration rate, *NOAC* non-vitamin K antagonist oral anticoagulant, *OAC* oral anticoagulant, *SBP* systolic blood pressure, *TTR* time in therapeutic range, *VKA*, vitamin K antagonist, QRISK-3, algorithm to calculate the risk of developing a heart attack or stroke over the next 10 years

Recommendations were classified as ‘ABC specific’ (relating to implementation of the ABC pathway for AF management) or ‘ABC non-specific’ (all other recommendations). General practitioners were contacted directly via email or via the practice manager and asked to review the recommendations and implement them at their discretion. A GP questionnaire was emailed one month after treatment recommendations were made to ascertain if they were implemented or not and the reason(s) why not. At six months follow-up, the researcher also accessed residents’ electronic medical records to establish if recommendations had been implemented. Treatment suggestions for complex patients were discussed with the wider multidisciplinary research team, including a consultant geriatrician, consultant cardiologist, health psychologist, and a senior pharmacist.

Residents in the usual care group continued to receive their usual care and followed their existing treatment plan. No changes were made to their medication as part of the study, but changes could still be made by their healthcare providers as part of usual care.

#### Outcome measures 

The primary outcome was a description of study feasibility. Feasibility outcomes included care home and resident recruitment and retention, in addition to completion rates of study questionnaires and assessments. This included GP questionnaires sent to ascertain the outcome(s) of pharmacist recommendation(s) (used alongside EMIS) and reason(s) for non-implementation if applicable, as well as resident self-report questionnaires to assess generic health-related quality of life (EuroQol-5-Dimensions-5-Levels questionnaire [EQ-5D-5L] [23, 24]) and disease-specific quality of life (Atrial Fibrillation Effect on Quality of Life questionnaire [AFEQT] [25]). In addition, researcher administered assessments requiring resident input to assess frailty (Edmonton Frail Scale – Acute Care [EFS-AC] [26–30]), cognitive function (six-item cognitive impairment test [6-CIT] [31]) and symptoms of AF (modified European Heart Rhythm Association [mEHRA] symptom scale [22]) and those completed independently by the researcher to assess stroke (CHA_2_DS_2_-VASc) [21] and bleeding risk (HAS-BLED [32]), resident frailty (Rockwood Clinical Frailty Scale [Rockwood CFS] [33], electronic Frailty Index [eFI] [34]) and resident level of dependency in activities of daily living (Barthel Index of Activities of Daily Living [35]) were noted. A list of study materials is provided in Supplement 1, Table S1.2.

Secondary outcomes were the number and type of pharmacist medication recommendations, GP implementation of recommendations, health events including ischaemic stroke, haemorrhagic stroke, systemic embolism, mortality, major bleeding (defined as fatal bleeding, symptomatic bleeding in a critical area or organ, or bleeding causing a fall in haemoglobin by ≥ 2 g/dL or a transfusion of ≥ 2 units of whole blood or red cells) [36], number of hospital admissions and number of falls.

#### Study schedule and data collection

The study schedule involved screening, baseline data collection and six month follow-up (Supplement 1, Table S1.3). Feasibility data on care home and resident recruitment were collected during screening. Electronic GP records (EMIS) and other medical records kept within care homes were used to obtain demographic, clinical and medication history at baseline and six months, as well as health-events occurring at any time point during the study period (Supplement 1, Table S1.2).

Questionnaire data (AFEQT and EQ-5D-5L) were collected by resident self-report during interview (over telephone, video call or face-to-face) with the researcher at baseline and six months. If a resident lacked capacity, the AFEQT questionnaire was omitted and a proxy version of the EQ-5D-5L was administered to the resident’s LPA for Health and Welfare over the telephone. Researcher-administered assessments requiring resident input (EFS-AC and 6-CIT) were carried out at baseline and six months over the telephone, video call or face-to-face. For the EFS-AC, all residents (with and without capacity) were asked all three items that could only be answered by the resident without any input from others. All other items were scored using the best available information obtained from interviews with carers and review of residents’ EMIS records. Symptom assessment using the mEHRA score was omitted in residents without capacity. The researcher asked carers about residents’ adherence to medication at baseline, and collected data on residents’ level of dependency in activities of daily living (Barthel Index) during interview (over telephone or face-to-face) at baseline and six months. All other data were collected by the researcher after independent assessment of the resident (Rockwood CFS), or review of EMIS records to calculate their eFI, CHA_2_DS_2_-VASc and HAS-BLED risk assessment scores at baseline and six months.

The number and type of pharmacist recommendations were recorded at baseline. Resident/care home retention, GP implementation of pharmacist recommendations and completion rate of questionnaires/assessments were collected at six months. Health-events and medication history were also collected at 12 months using EMIS records for all residents recruited before 1 July 2021 (Supplement 1, Table S1.2). The end of the trial was defined as follow-up of all residents recruited before 1 July 2021 for 12 months, and follow-up of all participants recruited after this date for six months. In the original study design, a 12 month follow-up was planned for all participants but because of significant delays in recruitment resulting from COVID-19, the recruitment period was extended and follow-up was shortened for those residents recruited after June 2021.

#### Statistics

Formal hypothesis testing was not conducted because this is not recommended in the CONSORT extension for pilot and feasibility studies [18]. Feasibility outcomes are reported using narrative synthesis and descriptive statistics are presented for relevant outcomes.

#### Patient and public involvement

The PIVOTALL research team engaged with care home managers and care home pharmacists who provided feedback that the study outcomes were of interest. Care home residents were not included.

## Results

### Primary outcome (study feasibility)

#### Recruitment of care homes and residents

**Fig. 1:**
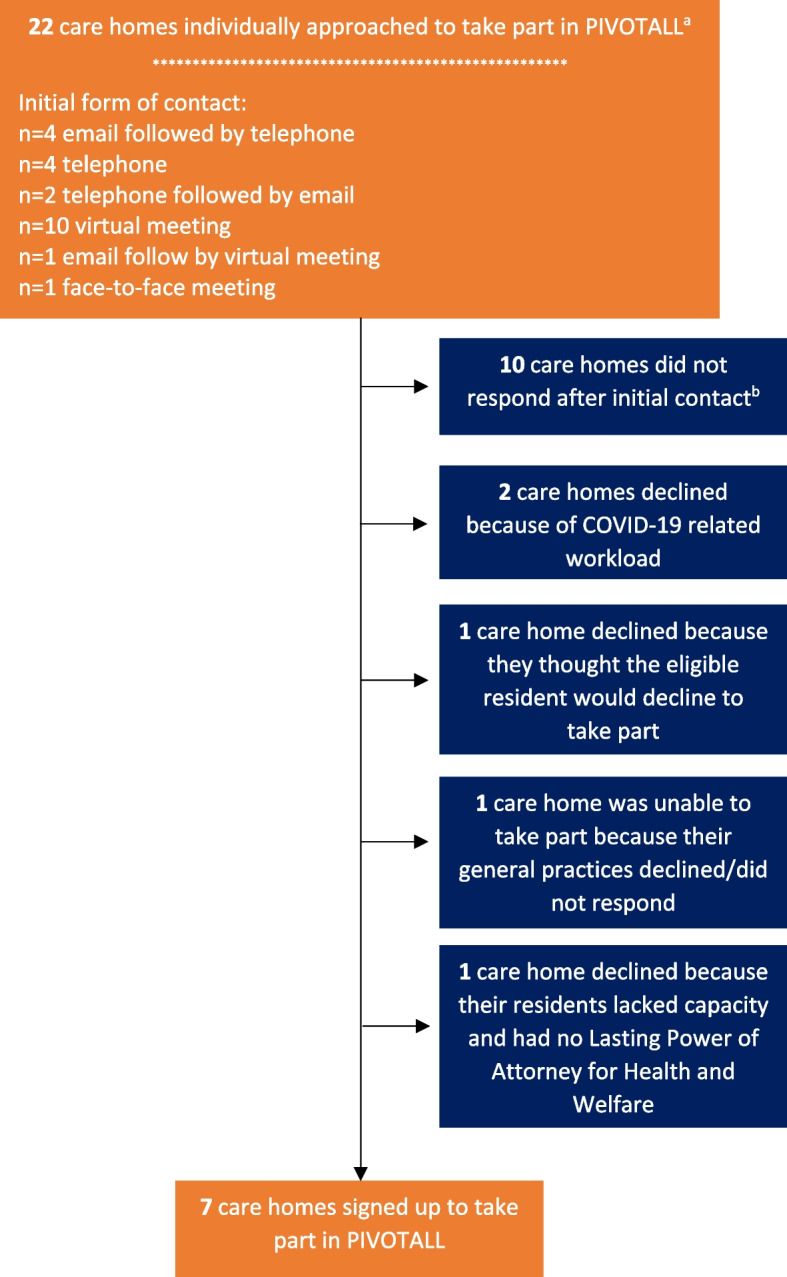
Flow diagram of care home recruitment. ^a^initial introduction to care home facilitated by Mersey Care NHS Foundation Trust medicines management pharmacists (*n* = 7), consultant geriatrician (*n* = 7), community matron (*n* = 3), general practice-based physician associate (*n* = 1), general practitioner (*n* = 1), care home manager of participating care home (*n* = 3). ^b^all care homes that did not respond were contacted at least once more

Twenty-two care homes were approached about the study, and seven agreed to take part between 28 September 2020 and 29 April 2021 (Fig. 1). Care home introductions were predominantly facilitated by a consultant geriatrician (AA) and medicines management pharmacists working within the care home setting (Fig. 1). For all recruited care homes, the manager made the decision to participate. Attempts were made to recruit further care homes by including a study summary in the North-West Coast Clinical Research Network care home newsletter in December 2020, but there were no expressions of interest. Researchers also attended a network meeting for general practices within the same locality in November 2020 and asked practices providing medical support to care homes to participate. Practices were supportive of the study but were unable to help due to COVID-19-related workload.

The time taken to recruit care homes was calculated according to the number of days between researchers first approaching the care home and the signing of the Site and PIC agreement by the care home manager. This ranged from 0 (care home signed up on day of initial contact by the researcher) to 122 days (mean 44.7 days [standard deviation, SD 48.7], median 22 days [interquartile range, IQR 3–88]). Time taken to obtain consent from general practices to act as PIC sites ranged from 0 (consent from general practice on day of initial contact by researchers) to 153 days (mean 50.9 days [SD 54.4], median 44 days [IQR 8–81]) between first approach by the researcher and signing of the PIC agreement.

**Fig. 2:**
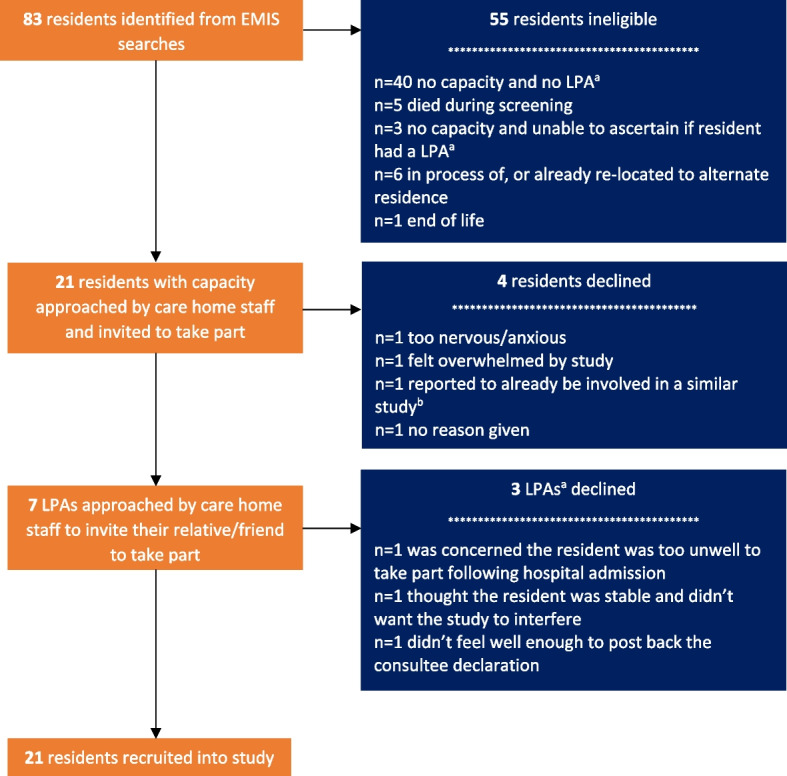
Flow diagram of participant recruitment. EMIS; Egton Medical Information Systems; LPA, Lasting Power of Attorney. ^a^LPA for Health and Welfare. ^b^details of study unknown

The study opened to resident recruitment on 13 October 2020 and closed on 31 November 2021. Across the seven participating care homes, 83 residents were identified from EMIS searches as potentially eligible (Fig. 2). The proportion of eligible residents identified from the search ranged between individual care homes from 11 to 100%. In total, 28 (33.7%) residents approached to participate either directly (*n* = 21), or via their LPA for Health and Welfare (for those without mental capacity to consent, *n* = 7) (Fig. 2). Most (*n* = 40) residents were ineligible because they did not have capacity and had no LPA for Health and Welfare (Fig. 2). Recruitment rates of eligible residents varied from 40 to 100% among care homes. Overall, 21 residents were recruited, less than the planned target recruitment minimum of 50 (Fig. 2). Eleven residents were randomly allocated to the intervention group and 10 to the usual care group. The characteristics of 21 residents enrolled into the study are described in Supplement 2, Tables S2.1–3. Residents in the intervention group were older than those in usual care group (mean age [SD] 85.0 [6.5] vs. 80.5 [6.9] years, median age [IQR] 87.0 [79.0–90.0] vs. 82.5 [74.5–85.8], respectively), but the proportion of females in both groups was similar (7/11 [63.6%] intervention, 6/10 [60.0%] usual care, respectively). Most (18/21, 85.7%) residents lived in general residential (*n* = 4 intervention, *n* = 4 usual care) or general nursing (*n* = 7 intervention, *n* = 3 usual care) homes, and on enrolment into the study 15/21 residents were classified as severely frail by the eFI.

#### Retention of care homes and residents

None of the participating care homes withdrew from the study. No residents asked to withdraw from the study, but three residents were lost to follow-up at six months because they died (n = 2) or moved residence (n = 1). Twelve month follow-up data was not collected for four residents who were recruited after 31 June 2021.

#### Completion rate of study questionnaires and assessments

Researcher-administered resident self-report questionnaires (EQ-5D-5L and AFEQT) had different completion rates at baseline. Both components of the EQ-5D-5L (five health dimensions and visual analogue scale) had a 100% completion rate when the researcher administered the questionnaire to 17 residents with capacity, and a proxy version of the questionnaire to the LPA for Health and Welfare of four residents without capacity. The AFEQT questionnaire was administered to 17 residents with capacity, and was completed by 16/17 (94.1%) residents. One resident was unable to answer questions in the treatment concern and treatment satisfaction domains. Care home staff administered the AFEQT questionnaire to one resident in the intervention group because the researcher was unsuccessful in their attempt to conduct a remote interview over the telephone due to poor signal and resident hearing difficulties. A face-to-face interview could not be arranged due to COVID-19 visiting restrictions. At six months, completion rates of both questionnaires were lower compared to baseline rates. Completion rates for the EQ-5D-5L and AFEQT were calculated for 18 and 14 residents, respectively, because three residents were lost to follow-up. The EQ-5D-5L five health dimensions was completed by 17/18 residents (94.4%) because one resident was unresponsive to questioning. The visual analogue scale was completed by 14/18 residents (77.8%) because the same resident remained unresponsive, and two residents and one LPA said they were unable to provide an answer. The AFEQT questionnaire was completed by 13/14 residents (92.9%).

**Fig. 3:**
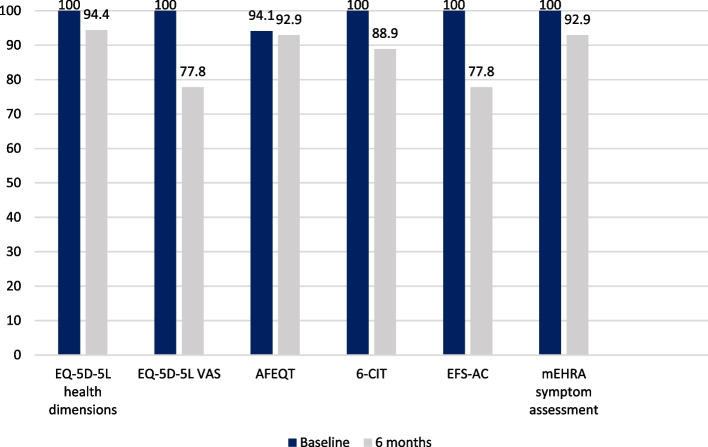
Completion rate of resident self-report researcher-administered questionnaires and researcher-administered assessments at baseline and six months. AFEQT, Atrial Fibrillation Effect on Quality of Life; 6-CIT, 6-item Cognitive Impairment Test; EFS-AC, Edmonton Frail Scale – Acute Care; EQ-5D-5L, EuroQol-5-Dimensions-5-Levels questionnaire; mEHRA, modified European Heart Rhythm Association; VAS, visual analogue scale

Researcher-administered assessments requiring resident input (6-CIT and EFS-AC) were completed by all residents at baseline. At six months, excluding the three residents who were lost to follow-up, completion rates reduced to 88.9% and 77.8%, respectively. One resident refused to do the 6-CIT assessment, and another resident was unresponsive to questioning. Four residents (one with capacity, three without capacity) were unable to answer one or more EFS-AC questions on general health status, social support or mood. The mEHRA symptom score was used to assess AF symptoms in all 17 residents with capacity at baseline. At six months, symptom assessment was carried out in 13 residents. Follow-up data were unavailable for four residents; one resident did not respond to questioning about symptoms at six month follow-up, two residents died and one resident moved residence. Completion rates of study questionnaires and assessments requiring resident input are reported in Fig. 3. There was 100% completion rate of all assessments conducted independently by the researcher at baseline and six months to assess stroke (CHA_2_DS_2_-VASc) and bleeding risk (HAS-BLED), resident frailty (Rockwood CFS, eFI) and resident level of dependency in activities of daily living (Barthel Index of Activities of Daily Living).

### Secondary outcomes

#### Pharmacist recommendations from medication review

**Fig. 4:**
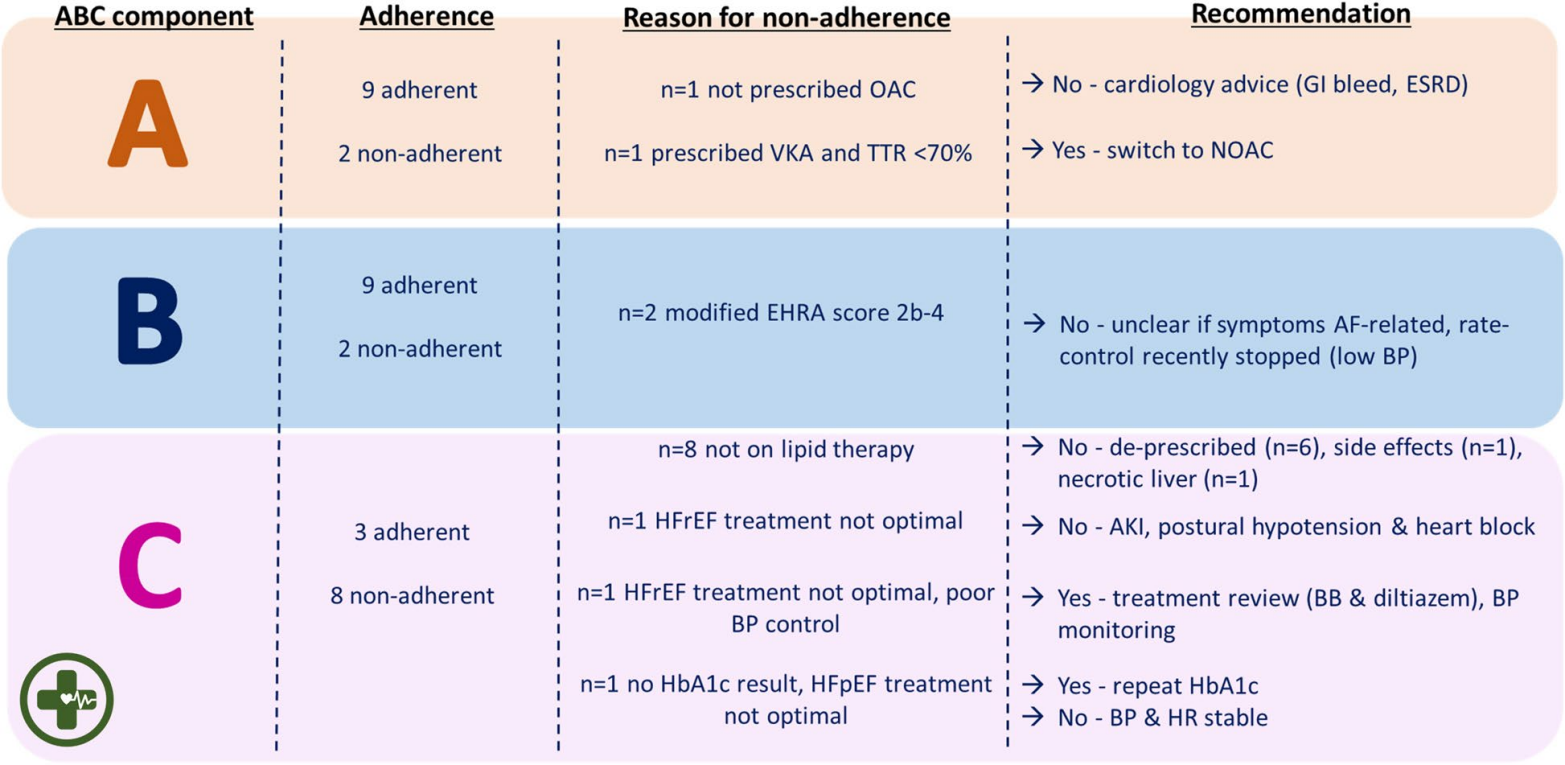
Adherence to the Atrial Fibrillation Better Care pathway and pharmacist recommendations in the event of non-adherence. AF, atrial fibrillation; ACEI, angiotensin converting enzyme inhibitor; AKI, acute kidney injury; BB, beta-blocker; BP, blood pressure; EHRA, European Heart Rhythm Association; ESRD, end stage renal disease; GI, gastrointestinal; HbA1c, glycated haemoglobin; HFpEF, heart failure with preserved ejection fraction (≥ 50%); HFrEF, heart failure with reduced ejection fraction (< 40%); HR, heart rate; LFTs, liver function tests; NOAC, non-vitamin K antagonist oral anticoagulant; OAC, oral anticoagulant; TTR, time in therapeutic range; VKA, vitamin K antagonist

**Fig. 5:**
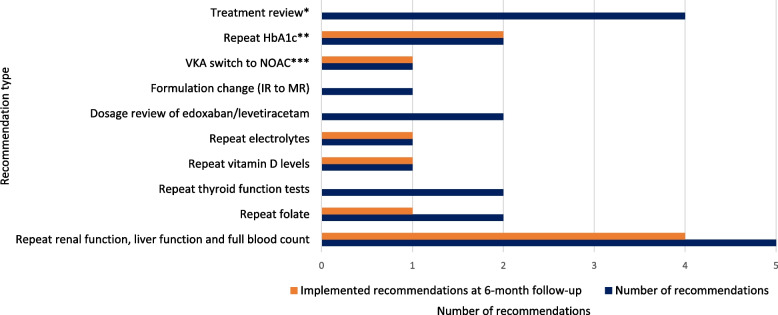
Number and type of all pharmacist medicines recommendations and implementation rates at six month follow-up. HbA1c, glycated haemoglobin; IR, immediate release; MR, modified release; NOAC, non-vitamin K antagonist oral anticoagulant; VKA, vitamin K antagonist. *two ABC-specific recommendations for one resident to (1) review diltiazem and bisoprolol in a resident with heart failure and then (2) review antihypertensive medications accordingly (readings > 140/85 mmHg), two non-ABC specific recommendations to rationalise bendroflumethiazide/perindopril in a resident with hypotension, and rationalise linagliptin in a diabetic resident with a HbA1c of 42 mmol/mol. **one ABC-specific recommendation to repeat HbA1c in a resident with documented history of type 2 diabetes but no record of blood glucose levels, HbA1c or prescription of antidiabetic medicines, one non-ABC specific recommendation to repeat HbA1c in a resident with type 2 diabetes who had gliclazide stopped with no repeat HbA1c. ***ABC-specific recommendation for a resident on warfarin with time in therapeutic range < 70%

Eleven residents in the intervention group received a pharmacist-led medication review. Three (of 11) residents were identified as fully adherent to all three components of the ABC pathway. When individual ABC pathway components were considered, adherence was higher to the ‘A’ (9/11 residents) and ‘B’ (9/11 residents) components compared to ‘C’ (3/11 residents). Despite low adherence rates, the pharmacist only made four ABC-specific recommendations for three residents, as follows: (1) switch vitamin K antagonist (VKA) to a non-vitamin K antagonist oral anticoagulant (NOAC) in a resident with TTR < 70%; (2) repeat blood test for glycated haemoglobin (HbA1c) in a resident with a documented history of type 2 diabetes but no prior records of blood glucose levels, HbA1c or evidence of prescription or oral antidiabetic medicines; (3) review prescription of diltiazem and atenolol due to potential for additive effects and worsening of heart failure in a resident who was complaining of increased breathlessness, and then (4) review antihypertensive medications accordingly in the same resident who had multiple blood pressure readings > 140/85 mmHg (Fig. 4). Two of these residents also had additional, non-ABC specific recommendations made to their GP (Fig. 5). Four other residents also had non-ABC specific medicine recommendations. Most recommendations (8/11) were blood tests for monitoring of prescribed medications (Fig. 5). Overall, the pharmacist made ABC and non-ABC specific recommendations as part of a medication review for 7/11 residents in the intervention group.

#### Implementation of pharmacist recommendations and completion of GP questionnaires

Implementation rates of pharmacist recommendations at six months were variable (Fig. 5). A total of 21 pharmacist recommendations (ABC-specific and non-ABC specific) were made and 10 were implemented (47.6%). All recommendations were implemented by residents’ GPs in 4/7 (57.1%) medication reviews. Seven questionnaires were sent to four GPs to ascertain the outcome of pharmacist medicines recommendations. Three GPs received two questionnaires (one for each resident where recommendations were made), and another GP received one questionnaire. Four (out of 7) questionnaires were completed by two GPs. Reasons for not implementing the recommendations were only provided by one GP; bloods had recently been requested therefore repetition was not required for renal function, liver function and full blood count for NOAC monitoring. The same GP chose not to implement the other recommendations: to repeat thyroid function tests, review the dose of edoxaban in line with the latest bloods for renal function, review concomitant prescription of diltiazem and atenolol and review antihypertensive medication. The reason provided was that there was no perceived benefit. At 12 month follow-up, it was noted that after the pharmacist recommendations were made the resident was referred to cardiology who advised the GP to stop diltiazem.

#### Health events

There were 10 adverse health events recorded within six months, seven in the usual care group and three in the intervention group. Two residents (one intervention and one usual care) died before six month follow-up. Between baseline and six month data collection, one resident in the intervention group was admitted to hospital with major extracranial bleeding. Four residents in the usual care group were hospitalised for non-cardiovascular (n = 3) and cardiovascular (fast-AF) (*n* = 1) causes (Supplement 2, Table S2.4**)**.

## Discussion

This is the first prospective study of ABC pathway implementation in a real-world population of older care home residents. The principal findings are: (i) it was feasible to use the ABC pathway as a framework for pharmacist medication review, but a ceiling-effect was observed whereby most residents’ medications were already optimised as much as possible according to the ABC pathway; (ii) implementation rates of pharmacist recommendations approximated 48%; (iii) overall ABC adherence rates did not change after pharmacist medication review, but adherence to ‘A’ increased (from 9/11 to 10/11 residents); (iv) there were procedural and system barriers that impacted recruitment of care homes and residents into the study; (v) it was difficult to assess resident quality of life, AF symptoms and frailty using the AFEQT questionnaire, mEHRA symptom scale and EFS-AC, respectively, and (vi) it was not possible to draw any conclusions on the effect of the intervention on health-related outcomes because overall ABC adherence did not change and the study was underpowered.

The small number of ABC recommendations (*n* = 4) made in this study is reassuring and suggests that most residents with AF in the intervention group were already receiving optimal AF care. Whilst only 3/11 residents were fully adherent to all three components of the ABC pathway, this was a result of active decisions not to treat in the context of multiple chronic conditions and risk of medication-related adverse effects after an individualised assessment of the net risk–benefit, rather than omissions in care. In this context, person-centric decisions including non-prescription and de-prescribing are part of medicines optimisation and refinement to the ABC pathway to reflect this would be useful to facilitate application in this population. Implementation of ‘C’ pathway components was most challenging; it was not possible to assess symptoms in residents without capacity, and most cardiovascular medications had previously been de-prescribed, or were inappropriate because of acute kidney injury, postural hypotension and falls risk. The ceiling-effect in medicines optimisation observed in this study may be a result of recent investments made by NHS England in pharmacy services to support care homes [14, 15].

There was a higher number of non-ABC specific recommendations (*n* = 17) made in the study, and 12 (70.6%) were suggestions to repeat blood tests for routine medication monitoring. This observation also raises the question whether under-performing of monitoring tests is common in the care home population, or whether this resulted from re-prioritisation of workload during COVID-19. The rate of implementation (10/21, 47.6%) of pharmacist recommendations (ABC and non-ABC) by GPs was similar to those reported in other interventional care home studies at 43% [37] and 58.1% [38]. In most cases, it was not possible to ascertain reasons for non-implementation but it is likely that the GP’s previous experiences with local pharmacy teams performing medications reviews for care home residents will have had an impact. In addition, if the GP was aware that a resident had a previous medication review by a pharmacist, then they may not have considered recommendations made as part of this study necessary to follow. The role of pharmacists across Europe varies extensively, and pharmacist prescribing is more typically part of clinical practice in the UK and Ireland compared to other European nations [39]. When pharmacists are performing structured medication reviews, it is important to establish a collaborative working model with the clinicians who are also responsible for patient care. Each profession must understand the other’s skills and responsibilities, and liability for prescribing must be clearly outlined if this is part of the pharmacist’s practice [40].

The COVID-19 pandemic had a deleterious impact on the recruitment of care homes and residents into this study. The pandemic was not the only challenge faced. There were other procedural (encountered before research starts), system (encountered during research) and resident-specific barriers that also impacted on the set-up and delivery of this study, that are well described in the literature [41]. A major procedural barrier was the documentation required to register care homes as research sites that delayed site initiation. The time taken to recruit care homes ranged from 0–122 days in this study and is similar to another UK feasibility study that used systematic and targeted methods of care home recruitment [42]. From 245 care homes approached, 13 (5.3%) were recruited. Time taken from initial care home visits to screening residents for eligibility was reported to range from 7–137 days [42]. Another UK care home study testing medication review plus person-centred care to improve care for residents with dementia reported a longer time to recruit five care homes (mean 237 days), and recruited 34 eligible residents in total [37]. In a systematic review and meta-analysis of pharmacist services in nursing homes [43], only two non-UK based European studies were identified [44, 45]. One non-randomised controlled study in Belgium tested the impact of pharmacist medication review on appropriateness of prescribing. It recruited two care homes (total 148 residents), but did not report time taken to recruit homes [45]. A randomised controlled study in Stockholm assessed the impact of pharmacist-led medication review specialising in clinical pharmacology and cardiology in 80 residents across nine care homes. Again, the time taken to recruit homes was not reported [44]. Recruitment of residents, particularly those without the capacity to consent, was another procedural barrier encountered. Suggestions to establish resident and representatives’ preparedness to be approached about research at the point of care home admission have been made, to remove the need for consent to be obtained by care home staff before subsequent approach by the research team [41].

Adults without capacity must be included in care home research in order for research outputs to be valuable and representative [46]. However, it was not possible to assess quality of life or AF symptoms using the AFEQT questionnaire and mEHRA symptom scale, respectively, in residents without capacity. Data collection using these measures in participants with capacity was extremely difficult; three residents were unable to distinguish if symptoms were AF-related, and eight did not know they had AF. In addition, questions related to physical activity were not applicable to any of the residents who were bed bound (*n* = 5) or had severely limited mobility (*n* = 12), so residents scored highly because they did not think it was their AF that was the limiting factor in them performing these activities. Application of the EFS-AC to assess frailty was also limited in residents without capacity; total scores could not be calculated due to one or more missing responses for questions that only the resident was allowed to answer. Collection of this type of data is critical in care home research; both quality of life (including activities of daily living, pain, mood and emotional health) and frailty are listed in the International Consortium for Health Outcomes set of person-centred outcome measures for older people, aimed to improve their lives [47].

### Strengths and limitations

This is the first prospective study testing implementation of the ABC pathway in older care home residents and the first to report on person-centred outcomes, including frailty and quality of life. The study was inclusive of different types of care homes that provide varying levels of assistance and support to residents, but may not be representative of all care home populations because it was restricted to Liverpool and Sefton, UK, and all residents were of white ethnicity. Not all residents from participating care homes were eligible for inclusion if more than one general practice provided medical care to residents in the same home (consent was sought only from the practice where most of the residents were registered), potentially resulting in selection bias. The workload associated with COVID-19 for GPs is also likely to have impacted implementation rates of pharmacist recommendations in this study. Qualitative insights from this study would have been useful to inform changes to the intervention, particularly in relation to the approach of integrated and collaborative working between pharmacists and GPs.

## Conclusions

The ABC pathway can be used as a framework by pharmacists for medication review in care home residents with AF. It must be tailored to the individual, even if this means that a decision is made not to implement one or more pathway components. The pathway serves as a reference point for pharmacists to consider guideline-adherent AF care and encourages them to make active decisions about AF management. This will help to prevent omissions in care. The study adds to existing literature on ABC pathway implementation in high risk cohorts. It also highlights the need for AF-specific quality of life measures to be developed and validated for care home residents. Wider implementation would provide an important insight into regional and national variation in the management of care home residents with AF.

### Supplementary Information

**Additional file 1: Supplement 1.** Supplementary methods. **Figure S1.1.** Flow diagram of care home and resident identification and recruitment. EMIS; Egton Medical Information Systems; GP, general practitioner; LPA, Lasting Power of Attorney. *LPA for Health and Welfare. **Contact was via telephone or video call if necessary due to COVID-19. Information sheets and consent/declaration forms were sent out via post if necessary due to COVID-19. **Table S1. 2.** List of study materials.

## Data Availability

The datasets generated and/or analyzed during the current study are not publicly available due to concerns of maintaining participant anonymity due to the small sample size but are available from the corresponding author on reasonable request.
