# Supplementary material for: Pharmacist-led intervention for older people with atrial fibrillation in long-term care (PIVOTALL study): a randomised pilot and feasibility study
Source: BMC Geriatr. 2024 Jan 16;24:64. doi: 10.1186/s12877-023-04527-4 (PMC10790530; doi:10.1186/s12877-023-04527-4)
Supplement: Supplementary file 1 — Additional file 1: Supplement 1. Supplementary methods. Figure S1.1. Flow diagram of care home and resident identification and recruitment. EMIS; Egton Medical Information Systems; GP, general practitioner; LPA, Lasting Power of Attorney. *LPA for Health and Welfare. **Contact was via telephone or video call if necessary due to COVID-19. Information sheets and consent/declaration forms were sent out via post if necessary due to COVID-19. Table S1. 2. List of study materials. [file 12877_2023_4527_MOESM1_ESM.docx]

**Supplementary Material**

**Pharmacist-led intervention for older people with atrial fibrillation in long-term care (PIVOTALL study): a randomised pilot and feasibility study**

^
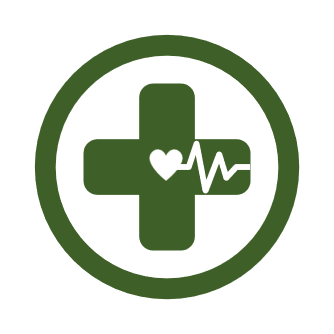
^

Leona A Ritchie*^1,2,3^ (PhD), Peter E Penson^1,3^ (PhD), Asangaedem Akpan^4,5^ (MPhil), Gregory Y H Lip^6,7^ (MD), Deirdre A Lane^1,2,7^ (PhD)

^1^Liverpool Centre for Cardiovascular Science, University of Liverpool, Liverpool, L7 8TX, United Kingdom

^2^Department of Cardiovascular and Metabolic Medicine, Institute of Life Course and Medical Sciences, University of Liverpool, Liverpool, L7 8TX, United Kingdom

^3^Clinical Pharmacy and Therapeutics Research Group, School of Pharmacy and Biomolecular Sciences, Liverpool John Moores University, Liverpool, L3 3AF, United Kingdom

^4^Musculoskeletal and Ageing Science, Institute of Life Course and Medical Sciences, University of Liverpool, L7 8TX, United Kingdom

^5^Liverpool University Hospitals NHS Foundation Trust, Liverpool, L9 7AL, United Kingdom

^6^Liverpool Centre for Cardiovascular Science, University of Liverpool, Liverpool John Moores University and Liverpool Heart and Chest Hospital, Liverpool, United Kingdom

^7^Danish Center for Health Services Research, Department of Clinical Medicine, Aalborg University, Aalborg, Denmark

***Correspondence to:** Leona A Ritchie, Liverpool Centre for Cardiovascular Science, William Henry Duncan Building, University of Liverpool, Liverpool, L7 8TX, United Kingdom.

Email: [leona.ritchie@liverpool.ac.uk](mailto:leona.ritchie@liverpool.ac.uk) Tel: 0151 706 4070 Fax: Not available

Table of contents

[Supplement 1. Supplementary methods. 3](#_Toc143244026)

[Supplement 2. Supplementary results. 7](#_Toc143244027)

# Supplement 1. Supplementary methods.


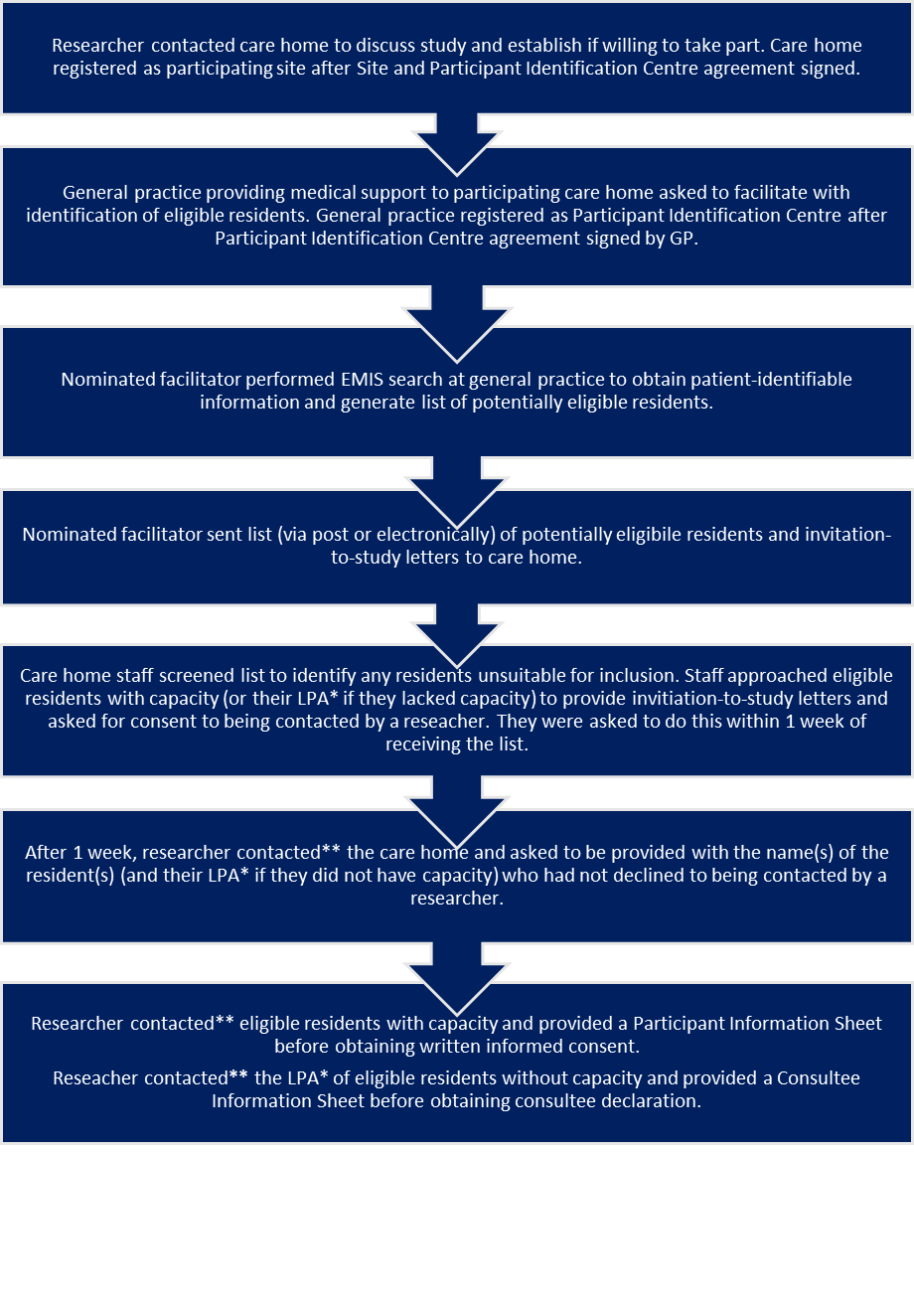


Figure S1.1. Flow diagram of care home and resident identification and recruitment.

EMIS; Egton Medical Information Systems; GP, general practitioner; LPA, Lasting Power of Attorney.

*LPA for Health and Welfare

**Contact was via telephone or video call if necessary due to COVID-19. Information sheets and consent/declaration forms were sent out via post if necessary due to COVID-19

**Table S1.2.** List of study materials.

| **Name:** | **Measure of:** |
| --- | --- |
| CHA_2_DS_2_-VASc assessment tool [1] | Stroke risk |
| HAS-BLED assessment tool [2] | Bleeding risk |
| Electronic Frailty Index [3] | Frailty |
| Edmonton Frail Scale – Acute Care [4-8] | Frailty |
| Rockwood Clinical Frailty Scale [9] | Frailty |
| Six-item Cognitive Impairment Test [10] | Cognition |
| EuroQol-5-Dimensions-5-Levels questionnaire [11, 12] | Generic health-related quality of life |
| Atrial Fibrillation Effect on Quality of Life questionnaire [13] | Atrial fibrillation-specific related quality of life |
| Barthel Index of Activities of Daily Living [14] | Dependency in activities of daily living |
| Modified European Heart Rhythm Association symptom scale [15] | Atrial fibrillation symptoms |

1. Lip GY, Nieuwlaat R, Pisters R, Lane DA, Crijns HJ, Lip GYH, et al. Refining clinical risk stratification for predicting stroke and thromboembolism in atrial fibrillation using a novel risk factor-based approach: the euro heart survey on atrial fibrillation. Chest. 2010;137(2):263-72.

2. Pisters R, Lane DA, Nieuwlaat R, de Vos CB, Crijns HJ, Lip GY. A novel user-friendly score (HAS-BLED) to assess 1-year risk of major bleeding in patients with atrial fibrillation: the Euro Heart Survey. Chest. 2010;138(5):1093-100.

3. Clegg A, Bates C, Young J, Ryan R, Nichols L, Ann Teale E, et al. Development and validation of an electronic frailty index using routine primary care electronic health record data. Age Ageing. 2016 May;45(3):353-60.

4. Fabricio-Wehbe SC, Schiaveto FV, Vendrusculo TR, Haas VJ, Dantas RA, Rodrigues RA. Cross-cultural adaptation and validity of the 'Edmonton Frail Scale - EFS' in a Brazilian elderly sample. Rev Lat Am Enfermagem. 2009 Nov-Dec;17(6):1043-9.

5. Fabrício-Wehbe S, Rosset I, Haas V, Diniz M, Spadoti D, Partezani-Rodrigues R. Reproducibility of the Brazilian version of the Edmonton Frail Scale for elderly living in the community. Rev Lat Am Enfermagem. 2013 10/16;21(6):1330-6.

6. Rolfson DB, Majumdar SR, Tsuyuki RT, Tahir A, Rockwood K. Validity and reliability of the Edmonton Frail Scale. Age Ageing. 2006 Sep;35(5):526-9.

7. Ramirez Ramirez JU, Cadena Sanabria MO, Ochoa ME. Edmonton Frail Scale in Colombian older people. Comparison with the Fried criteria. Rev Esp Geriatr Gerontol. 2017 Nov - Dec;52(6):322-5.

8. Rolfson DB. Edmonton Frail Scale. 2020 [cited 2020 Sept 11]. Available from: <https://edmontonfrailscale.org/frailty-covid-19>.

9. Rockwood K, Song X, MacKnight C, Bergman H, Hogan DB, McDowell I, et al. A global clinical measure of fitness and frailty in elderly people. CMAJ. 2005;173(5):489-95.

10. Katzman R, Brown T, Fuld P, Peck A, Schechter R, Schimmel H. Validation of a short Orientation-Memory-Concentration Test of cognitive impairment. Am J Psychiatry. 1983 Jun;140(6):734-9.

11. EuroQol Group. EuroQol--a new facility for the measurement of health-related quality of life. Health policy (Amsterdam, Netherlands). 1990 Dec;16(3):199-208.

12. Brooks R. EuroQol: the current state of play. Health policy (Amsterdam, Netherlands). 1996 Jul;37(1):53-72.

13. Spertus J, Dorian P, Bubien R, Lewis S, Godejohn D, Reynolds MR, et al. Development and validation of the Atrial Fibrillation Effect on QualiTy-of-Life (AFEQT) Questionnaire in patients with atrial fibrillation. Circ Arrhythm Electrophysiol. 2011 Feb;4(1):15-25.

14. Mahoney FI, Barthel DW. Functional evaluation: The Barthel Index. Md State Med J. 1965 Feb;14:61-5.

15. Wynn GJ, Todd DM, Webber M, Bonnett L, McShane J, Kirchhof P, et al. The European Heart Rhythm Association symptom classification for atrial fibrillation: validation and improvement through a simple modification. Europace. 2014;16(7):965-72.

| Table S1.3. Study schedule and time-points for data collection. | | | | | |
| --- | --- | --- | --- | --- | --- |
| **Procedures** | ***** | **Study timeline** | | | |
|  |  | Screening | Baseline | 6 months | 12 months^d^ |
| Care home recruitment rate |  | X |  |  |  |
| Review inclusion/exclusion criteria |  | X |  |  |  |
| Written consent/consultee declaration |  | X |  |  |  |
| Resident recruitment rate |  | X |  |  |  |
| Care home retention rate |  |  |  | X | X |
| Resident retention rate |  |  |  | X | X |
| Resident demographics |  |  | X |  |  |
| Medical history |  |  | X |  |  |
| Medication history |  |  | X | X | X |
| Allocation to intervention or usual care |  |  | X |  |  |
| Pharmacist medication review^a^ |  |  | X |  |  |
| Pharmacist medicine recommendations^a^ |  |  | X |  |  |
| Implementation of recommendations^a^ |  |  |  | X |  |
| GP questionnaire^a^ |  |  |  | X |  |
| Completion rate of GP questionnaire^a^ |  |  |  | X |  |
| AFEQT^b^ |  |  | X | X |  |
| EQ-5D-5L^c^ |  |  | X | X |  |
| Edmonton Frail Scale Acute Care |  |  | X | X |  |
| 6-item Cognitive Impairment Test |  |  | X | X |  |
| Rockwood Clinical Frailty Scale |  |  | X | X |  |
| Electronic Frailty Index |  |  | X | X |  |
| Barthel Index of Activities of Daily Living |  |  | X | X |  |
| CHA_2_DS_2_-VASc (stroke risk) |  |  | X | X |  |
| HAS-BLED (bleeding risk) |  |  | X | X |  |
| Completion rate of questionnaires |  |  | X | X |  |
| Completion rate of assessments |  |  | X | X |  |
| AFEQT, Atrial Fibrillation Effect on Quality of Life questionnaire; EQ-5D-5L, EuroQol-5-Dimensions-5-Levels; GP, general practitioner.  ^a^procedures relevant to intervention group only; ^b^omitted in residents without capacity; ^c^completed by Lasting Power of Attorney for Health and Welfare for residents without capacity; ^d^12 month follow-up for residents recruited before 1 July 2021   \| ***Key – procedure completed by:** \| \| \| --- \| --- \| \| Research pharmacist \| Resident (self-reported researcher-administered questionnaire) \| \| General practitioner \| Research pharmacist with resident input (researcher-administered assessment) \| | | | | | |

# Supplement 2. Supplementary results.

Table S2.1. Baseline demographics, frailty, stroke and bleeding risk, social history and level of dependency of care home residents on entry into study.

| **Resident characteristics, n (%)** | **All residents (n=21)** | **Usual care (n=10)** | **Intervention (n=11)** |
| --- | --- | --- | --- |
| **Demographics** |  |  |  |
| Age (mean [SD]) | 82.9 [6.9] | 80.5 [6.9] | 85.0 [6.5] |
| Age (median [IQR]) | 83.0  [77.5-88.0] | 82.5  [74.5-85.8] | 87.0  [79.0-90.0] |
| Age categories |  |  |  |
| 65-74 years | 3 (14.3) | 2 (20.0) | 1 (9.1) |
| 75-84 years | 8 (38.1) | 4 (40.0) | 4 (36.4) |
| 85-94 years | 9 (42.9) | 4 (40.0) | 5 (45.5) |
| >=95 years | 1 (4.8) | 0 (0.0) | 1 (9.1) |
| Female | 13 (61.9) | 6 (60.0) | 7 (63.6) |
| Ethnicity |  |  |  |
| White (English/Welsh/Scottish/Northern Irish/British) | 21 (100.0) | 10 (100.0) | 11 (100.0) |
| Mixed/Multiple ethnic groups | 0 (0.0) | 0 (0.0) | 0 (0.0) |
| Asian/Asian British | 0 (0.0) | 0 (0.0) | 0 (0.0) |
| Black/African/Caribbean/Black British | 0 (0.0) | 0 (0.0) | 0 (0.0) |
| Other ethnic group | 0 (0.0) | 0 (0.0) | 0 (0.0) |
| Type of care home |  |  |  |
| General residential | 8 (38.1) | 4 (40.0) | 4 (36.4) |
| General nursing | 10 (47.6) | 3 (30.0) | 7 (63.6) |
| Nursing EMI | 1 (4.8) | 1 (10.0) | 0 (0.0) |
| Residential EMI | 2 (9.5) | 2 (20.0) | 0 (0.0) |
| Weight |  |  |  |
| <50kg | 4 (19.0) | 2 (20.0) | 2 (18.2) |
| 50-99kg | 14 (66.7) | 7 (70.0) | 7 (63.6) |
| 100-149kg | 3 (14.3) | 1 (10.0) | 2 (18.2) |
| >=150kg | 0 (0.0) | 0 (0.0) | 0 (0.0) |
| **Frailty** |  |  |  |
| Electronic Frailty Index |  |  |  |
| No frailty | 0 (0.0) | 0 (0.0) | 0 (0.0) |
| Mild | 3 (14.3) | 2 (20.0) | 1 (9.1) |
| Moderate | 3 (14.3) | 1 (10.0) | 2 (18.2) |
| Severe | 15 (71.4) | 7 (70.0) | 8 (72.7) |
| **Stroke and bleeding risk^a^** |  |  |  |
| Stroke risk (CHA_2_DS_2_-VASc) |  |  |  |
| Score (mean [SD]) | 5.1 [1.7] | 4.9 [1.9] | 5.4 [1.4] |
| Score (median [IQR]) | 5.0 [4.0-6.0] | 5.0 [3.8-6.0] | 6.0 [4.0-7.0] |
| Moderate risk (score 1 males) | 1 (4.8) | 1 (10.0) | 0 (0.0) |
| High risk (score ≥2 males and females) | 20 (95.2) | 9 (90.0) | 11 (100.0) |
| Bleeding risk (HAS-BLED score) |  |  |  |
| Score (mean [SD]) | 1.7 [0.9] | 1.8 [0.9] | 1.5 [0.9] |
| Score (median [IQR]) | 1.0 [1.0-2.0] | 1.5 [1.0-3.0] | 1.0 [1.0-2.0] |
| Low risk (score 0-2) | 17 (81.0) | 7 (70.0) | 10 (90.9) |
| High risk (score ≥3) | 4 (19.0) | 3 (30.0) | 1 (9.1) |
| **Social history** |  |  |  |
| Smoking |  |  |  |
| Current | 1 (4.8) | 0 (0.0) | 1 (9.1) |
| Ex | 10 (47.6) | 7 (70.0) | 3 (27.3) |
| Never | 10 (47.6) | 3 (30.0) | 7 (63.6) |
| Alcohol use |  |  |  |
| Never | 15 (71.4) | 8 (80.0) | 7 (63.6) |
| <1 drink/week | 3 (14.3) | 1 (10.0) | 2 (18.2) |
| 1-2 drinks/week | 2 (9.5) | 1 (10.0) | 1 (9.1) |
| 3-4 drinks/week | 0 (0.0) | 0 (0.0) | 0 (0.0) |
| 5-6 drinks/week | 0 (0.0) | 0 (0.0) | 0 (0.0) |
| 7-8 drinks/week | 1 (4.8) | 0 (0.0) | 1 (9.1) |
| >8 drinks/week | 0 (0.0) | 0 (0.0) | 0 (0.0) |
| **Dependence in activities of daily living** |  |  |  |
| Barthel Index |  |  |  |
| Independent | 3 (14.3) | 2 (20.0) | 1 (9.1) |
| Minimally dependent | 3 (14.3) | 3 (30.0) | 0 (0.0) |
| Partially dependent | 5 (23.8) | 2 (20.0) | 3 (27.3) |
| Very dependent | 6 (28.6) | 1 (10.0) | 5 (45.5) |
| Totally dependent | 4 (19.0) | 2 (20.0) | 2 (18.2) |

EMI, elderly mental infirm; IQR, interquartile range; SD, standard deviation.

^a^no residents were categorised as low risk of stroke (CHA_2_DS_2_-VASc score 0 males, score 1 females) because all residents were aged ≥65 years

Table S2.2. Baseline cardiovascular and non-cardiovascular medical history of care home residents on entry into study.

| **Medical history, n (%)** | **All residents (n=21)** | **Usual care (n=10)** | **Intervention (n=11)** |
| --- | --- | --- | --- |
| **Cardiovascular disease** |  |  |  |
| Atrial fibrillation |  |  |  |
| First-diagnosed | 0 (0.0) | 0 (0.0) | 0 (0.0) |
| Paroxysmal | 9 (42.9) | 6 (60.0) | 3 (27.3) |
| Persistent | 0 (0.0) | 0 (0.0) | 0 (0.0) |
| Long-standing | 0 (0.0) | 0 (0.0) | 0 (0.0) |
| Permanent | 2 (9.5) | 1 (10.0) | 1 (9.1) |
| Unclassified | 10 (47.6) | 3 (30.0) | 7 (63.6) |
| Hypertension | 18 (85.7) | 8 (80.0) | 10 (90.9) |
| Heart failure | 7 (33.3) | 4 (40.0) | 3 (27.3) |
| Coronary artery disease^a^ | 8 (38.1) | 5 (50.0) | 3 (27.3) |
| Valvular heart disease | 9 (42.9) | 5 (50.0) | 4 (36.4) |
| Cardiomyopathy | 2 (9.5) | 1 (10.0) | 1 (9.1) |
| Carotid disease | 1 (4.8) | 1 (10.0) | 0 (0.0) |
| Pulmonary hypertension | 2 (9.5) | 1 (10.0) | 1 (9.1) |
| Intracranial haemorrhage | 2 (9.5) | 2 (20.0) | 0 (0.0) |
| Ischaemic stroke | 3 (14.3) | 0 (0.0) | 3 (27.3) |
| Transient ischaemic attack | 0 (0.0) | 0 (0.0) | 0 (0.0) |
| Major extracranial bleeding | 2 (9.5) | 1 (10.0) | 1 (9.1) |
| Peripheral vascular disease | 1 (4.8) | 1 (10.0) | 0 (0.0) |
| Venous thromboembolism | 7 (33.3) | 3 (30.0) | 4 (36.4) |
| **Endocrine disease** |  |  |  |
| Thyroid disease | 5 (23.8) | 1 (10.0) | 4 (36.4) |
| Parathyroid disease | 1 (4.8) | 1 (10.0) | 0 (0.0) |
| Type 1 diabetes mellitus | 0 (0.0) | 0 (0.0) | 0 (0.0) |
| Type 2 diabetes mellitus | 8 (38.1) | 4 (40.0) | 4 (36.4) |
| **Respiratory disease** |  |  |  |
| Asthma | 2 (9.5) | 2 (20.0) | 0 (0.0) |
| Chronic obstructive pulmonary disease | 4 (19.0) | 3 (30.0) | 1 (9.1) |
| Emphysema | 1 (4.8) | 0 (0.0) | 1 (9.1) |
| Bronchitis | 3 (14.3) | 1 (10.0) | 2 (18.2) |
| **Gastrointestinal disease** |  |  |  |
| Crohn’s disease | 0 (0.0) | 0 (0.0) | 0 (0.0) |
| Ulcerative colitis | 2 (9.5) | 1 (10.0) | 1 (9.1) |
| Irritable bowel syndrome | 2 (9.5) | 1 (10.0) | 1 (9.1) |
| Dyspepsia | 9 (42.9) | 6 (60.0) | 3 (27.3) |
| Hiatus hernia | 4 (19.0) | 2 (20.0) | 2 (18.2) |
| Diverticular disease | 3 (14.3) | 2 (20.0) | 1 (9.1) |
| Hepatitis | 0 (0.0) | 0 (0.0) | 0 (0.0) |
| Cirrhosis | 0 (0.0) | 0 (0.0) | 0 (0.0) |
| Portal hypertension | 0 (0.0) | 0 (0.0) | 0 (0.0) |
| Oesophageal varices | 0 (0.0) | 0 (0.0) | 0 (0.0) |
| **Blood disorders** |  |  |  |
| Anaemia | 8 (38.1) | 2 (20.0) | 6 (54.5) |
| **Mental, behavioural, neurodegenerative, neurodevelopmental disorders** | | | |
| Alzheimer’s disease | 3 (14.3) | 2 (20.0) | 1 (9.1) |
| Vascular dementia | 0 (0.0) | 0 (0.0) | 0 (0.0) |
| Frontotemporal dementia | 0 (0.0) | 0 (0.0) | 0 (0.0) |
| Schizophrenia | 0 (0.0) | 0 (0.0) | 0 (0.0) |
| Parkinson’s disease | 0 (0.0) | 0 (0.0) | 0 (0.0) |
| Epilepsy | 0 (0.0) | 0 (0.0) | 0 (0.0) |
| Anxiety | 3 (14.3) | 1 (10.0) | 2 (18.2) |
| Bipolar disorder | 1 (4.8) | 1 (10.0) | 0 (0.0) |
| Major depression^b^ | 3 (14.3) | 1 (10.0) | 2 (18.2) |
| Depressive symptomatology | 7 (33.3) | 4 (40.0) | 3 (27.3) |
| **Ear, nose and throat disease** | | | |
| Meniere’s disease | 0 (0.0) | 0 (0.0) | 0 (0.0) |
| Tinnitus | 1 (4.8) | 0 (0.0) | 1 (9.1) |
| Sinusitis | 2 (9.5) | 1 (10.0) | 1 (9.1) |
| Rhinitis | 0 (0.0) | 0 (0.0) | 0 (0.0) |
| Nasal polyps | 0 (0.0) | 0 (0.0) | 0 (0.0) |
| **Skin disease** |  |  |  |
| Psoriasis | 1 (4.8) | 1 (10.0) | 0 (0.0) |
| Eczema | 2 (9.5) | 1 (10.0) | 1 (9.1) |
| Dermatitis | 3 (14.3) | 2 (20.0) | 1 (9.1) |
| **Eye disease** |  |  |  |
| Glaucoma | 4 (19.0) | 1 (10.0) | 3 (27.3) |
| Macular degeneration | 2 (9.5) | 1 (10.0) | 1 (9.1) |
| Cataracts | 4 (19.0) | 2 (20.0) | 2 (18.2) |
| Diabetic retinopathy | 1 (4.8) | 1 (10.0) | 0 (0.0) |
| **Urogenital disease** |  |  |  |
| Urinary tract infections | 17 (81.0) | 9 (90.0) | 8 (72.7) |
| Urinary retention | 4 (19.0) | 1 (10.0) | 3 (27.3) |
| Urinary incontinence | 7 (33.3) | 4 (40.0) | 3 (27.3) |
| Benign prostatic hyperplasia | 2 (9.5) | 1 (10.0) | 1 (9.1) |
| **Musculoskeletal disease** |  |  |  |
| Rheumatoid arthritis | 2 (9.5) | 0 (0.0) | 2 (18.2) |
| Osteoarthritis | 13 (61.9) | 5 (50.0) | 8 (72.7) |
| Polymyalgia rheumatica | 2 (9.5) | 1 (10.0) | 1 (9.1) |
| Fibromyalgia | 1 (4.8) | 1 (10.0) | 0 (0.0) |
| Gout | 3 (14.3) | 1 (10.0) | 2 (18.2) |
| **Renal disease** |  |  |  |
| Previous acute kidney injury | 5 (23.8) | 2 (20.0) | 3 (27.3) |
| Chronic kidney disease^c^ | 12 (57.1) | 4 (40.0) | 8 (72.7) |
| Dialysis | 1 (4.8) | 1 (10.0) | 0 (0.0) |
| Transplantation | 0 (0.0) | 0 (0.0) | 0 (0.0) |
| Renal vein thrombosis | 0 (0.0) | 0 (0.0) | 0 (0.0) |
| **Malignancy** (current/previous) | 6 (33.3) | 2 (20.0) | 4 (36.4) |

^a^including myocardial infarction, coronary artery bypass graft and angina; ^b^Diagnostic and Statistical Manual diagnosis; ^c^stage 2 (eGFR 60-89 ml/min/1.73 m^2^) n=1, stage 3a (eGFR 45-59 ml/min/1.73 m^2^) n=7, stage 3b (eGFR 30-44 ml/min/1.73 m^2^) n=2, stage 4 (eGFR 15-29 ml/min/1.73 m^2^) n=1, stage 5 (eGFR <15 ml/min/1.73 m^2^) n=1

Table S2.3. Baseline cardiovascular and non-cardiovascular medication history of care home residents on entry into study.

| **Medication history, n (%)** | **All residents (n=21)** | **Usual care (n=10)** | **Intervention (n=11)** |
| --- | --- | --- | --- |
| **Cardiovascular medications** |  |  |  |
| Non-vitamin K antagonist oral anticoagulants |  |  |  |
| Apixaban | 7 (33.3) | 2 (20.0) | 5 (45.5) |
| Rivaroxaban | 1 (4.8) | 0 (0.0) | 1 (9.1) |
| Dabigatran | 1 (4.8) | 0 (0.0) | 1 (9.1) |
| Edoxaban | 5 (23.8) | 3 (30.0) | 2 (18.2) |
| Vitamin K antagonists | 1 (4.8) | 0 (0.0) | 1 (9.1) |
| Antiplatelets | 1 (4.8) | 1 (10.0) | 0 (0.0) |
| Beta-blockers | 14 (66.7) | 8 (80.0) | 6 (54.5) |
| Rate-limiting calcium-channel blockers | 1 (4.8) | 0 (0.0) | 1 (9.1) |
| Cardiac glycosides | 1 (4.8) | 1 (10.0) | 0 (0.0) |
| Anti-arrhythmics | 0 (0.0) | 0 (0.0) | 0 (0.0) |
| Angiotensin converting enzyme inhibitors | 2 (9.5) | 1 (10.0) | 0 (0.0) |
| Angiotensin receptor blockers | 1 (4.8) | 0 (0.0) | 1 (9.1) |
| Dihydropyridine calcium channel blockers | 2 (9.5) | 0 (0.0) | 2 (18.2) |
| Alpha blockers | 0 (0.0) | 0 (0.0) | 0 (0.0) |
| Centrally-acting antihypertensives | 0 (0.0) | 0 (0.0) | 0 (0.0) |
| Nitrates | 3 (14.3) | 1 (10.0) | 2 (18.2) |
| Thiazide or thiazide-like diuretics | 1 (4.8) | 0 (0.0) | 1 (9.1) |
| Loop diuretics | 7 (33.3) | 3 (30.0) | 4 (36.4) |
| Potassium sparing diuretics | 0 (0.0) | 0 (0.0) | 0 (0.0) |
| Aldosterone receptor antagonists | 3 (14.3) | 1 (10.0) | 2 (18.2) |
| Statins | 7 (33.3) | 4 (40.0) | 3 (27.3) |
| Fibrates | 1 (4.8) | 0 (0.0) | 1 (9.1) |
| Other lipid lowering therapy^a^ | 1 (4.8) | 1 (10.0) | 0 (0.0) |
| Oral antidiabetic drugs^b^ | 4 (19.0) | 2 (20.0) | 2 (18.2) |
| Insulin | 0 (0.0) | 0 (0.0) | 0 (0.0) |
| **Non-cardiovascular medications** |  |  |  |
| Inhaled preparations^c^ | 4 (19.0) | 2 (20.0) | 2 (18.2) |
| Topical preparations^d^ | 19 (90.5) | 9 (90.0) | 10 (90.9) |
| Anti-emetics | 0 (0.0) | 0 (0.0) | 0 (0.0) |
| Substance dependence therapies | 0 (0.0) | 0 (0.0) | 0 (0.0) |
| Anti-migraine drugs | 0 (0.0) | 0 (0.0) | 0 (0.0) |
| Anti-Parkinson drugs | 0 (0.0) | 0 (0.0) | 0 (0.0) |
| Anxiolytics and hypnotics | 2 (9.5) | 1 (10.0) | 1 (9.1) |
| Supplements/replacement therapies^e^ | 17 (81.0) | 7 (70.0) | 10 (90.9) |
| Bisphosphonates | 2 (9.5) | 1 (10.0) | 1 (9.1) |
| Opioid analgesics | 6 (28.6) | 2 (20.0) | 4 (36.4) |
| Non-opioid analgesics | 14 (66.7) | 4 (40.0) | 10 (90.9) |
| Anti-epileptics | 1 (4.8) | 0 (0.0) | 1 (9.1) |
| Anti-psychotics and mood stabilising drugs | 2 (9.5) | 1 (10.0) | 1 (9.1) |
| Non-steroidal anti-inflammatory drugs | 0 (0.0) | 0 (0.0) | 0 (0.0) |
| Antacids | 2 (9.5) | 1 (10.0) | 1 (9.1) |
| Histamine-2 receptor antagonists | 0 (0.0) | 0 (0.0) | 0 (0.0) |
| Proton pump inhibitors | 14 (66.7) | 6 (60.0) | 8 (72.7) |
| Benign prostatic hyperplasia drugs | 2 (9.5) | 1 (10.0) | 1 (9.1) |
| Thyroid hormones | 5 (23.8) | 0 (0.0) | 4 (36.4) |
| Antithyroid drugs | 0 (0.0) | 0 (0.0) | 0 (0.0) |
| Parathyroid hormones | 0 (0.0) | 0 (0.0) | 0 (0.0) |
| Antiparathyroid hormones | 0 (0.0) | 0 (0.0) | 0 (0.0) |
| Corticosteroids | 0 (0.0) | 0 (0.0) | 0 (0.0) |
| Endocrine therapies | 0 (0.0) | 0 (0.0) | 0 (0.0) |
| Antidepressants | 9 (42.9) | 3 (30.0) | 6 (54.5) |
| Antihistamines | 1 (4.8) | 0 (0.0) | 1 (9.1) |
| Antispasmodics | 1 (4.8) | 0 (0.0) | 1 (9.1) |
| Biological therapy | 0 (0.0) | 0 (0.0) | 0 (0.0) |
| Laxatives | 12 (57.1) | 4 (40.0) | 8 (72.7) |
| Urinary incontinence/frequency/urgency drugs | 3 (14.3) | 2 (20.0) | 1 (9.1) |
| Anti-diarrhoeal drugs | 1 (4.8) | 0 (0.0) | 1 (9.1) |
| Aminosalicylates | 2 (9.5) | 1 (10.0) | 1 (9.1) |
| Immunosuppressive therapies | 1 (4.8) | 0 (0.0) | 1 (9.1) |
| Antidementia drugs | 3 (14.3) | 2 (20.0) | 1 (9.1) |
| Anticramping drugs | 1 (4.8) | 1 (10.0) | 0 (0.0) |
| Muscle relaxants | 0 (0.0) | 0 (0.0) | 0 (0.0) |

^a^ezetimibe

^b^metformin immediate release n=2, linagliptin n=2

^c^including inhalers, nebules, oxygen therapy

^d^including ear/eye drops, creams/ointments/lotions, nasal sprays, medicated shampoos, bath additives, any other topical preparation

^e^including iron, calcium, vitamins, electrolytes, enzymes

Table S2.4 Number and type of adverse health events in residents at follow-up.

| **Adverse health events** | **6 months** | | **12 months** | |
| --- | --- | --- | --- | --- |
|  | **Usual care (n=9^a^)** | **Intervention (n=9^b^)** | **Usual care (n=7^a^)** | **Intervention**  **(n=6^b^)** |
| Stroke (ischaemic/haemorrhagic) | 0 | 0 | 0 | 0 |
| Major extracranial bleeding^c^ | 0 | 1 | 0 | 0 |
| Mortality | 1 | 1 | 0 | 0 |
| Systemic embolism | 0 | 0 | 0 | 0 |
| Hospital visit(s)^d^ | 4 | 1 | 0 | 1 |
| Fall(s) | 2 | 0 | 0 | 0 |

^a^6 and 12 month follow-up data unavailable for one resident who died, no 12 month follow-up for two residents recruited after 31 June 2021

^b^6 and 12 month follow-up data unavailable for one resident who died and one resident who moved residence, no 12 month follow-up for two residents recruited after 31 June 2021 and one resident who moved residence

^c^fatal bleeding, symptomatic bleeding in a critical area or organ, or bleeding causing a fall in haemoglobin by ≥ 2g/dL or a transfusion of ≥ 2 units of whole blood or red cells^398^

^d^any unplanned hospital visits, including visits that did not result in hospital admission
